# Design of virtual BCI channels based on informer

**DOI:** 10.3389/fnhum.2023.1150316

**Published:** 2023-04-24

**Authors:** Hang Sun, Changsheng Li, He Zhang

**Affiliations:** Department of Mechanical Engineering, Nanjing University of Science and Technology School, Nanjing, Jiangsu, China

**Keywords:** electroencephalogram, informer, brain-computer interface (BCI), virtual channel, motor imagery

## Abstract

The precision and reliability of electroencephalogram (EEG) data are essential for the effective functioning of a brain-computer interface (BCI). As the number of BCI acquisition channels increases, more EEG information can be gathered. However, having too many channels will reduce the practicability of the BCI system, raise the likelihood of poor-quality channels, and lead to information misinterpretation. These issues pose challenges to the advancement of BCI systems. Determining the optimal configuration of BCI acquisition channels can minimize the number of channels utilized, but it is challenging to maintain the original operating system and accommodate individual variations in channel layout. To address these concerns, this study introduces the EEG-completion-informer (EC-informer), which is based on the Informer architecture known for its effectiveness in time-series problems. By providing input from four BCI acquisition channels, the EC-informer can generate several virtual acquisition channels to extract additional EEG information for analysis. This approach allows for the direct inheritance of the original model, significantly reducing researchers’ workload. Moreover, EC-informers demonstrate strong performance in damaged channel repair and poor channel identification. Using the Informer as a foundation, the study proposes the EC-informer, tailored to BCI requirements and demanding only a small number of training samples. This approach eliminates the need for extensive computing units to train an efficient, lightweight model while preserving comprehensive information about target channels. The study also confirms that the proposed model can be transferred to other operators with minimal loss, exhibiting robust applicability. The EC-informer’s features enable original BCI devices to adapt to a broader range of classification algorithms and relax the operational requirements of BCI devices, which could facilitate the promotion of the use of BCI devices in daily life.

## 1. Introduction

A brain-computer interface (BCI) is a communication system that operates independently of the brain’s typical output pathways, such as peripheral nerves and muscles (Wolpaw et al., 2000). A BCI enables direct connections between the human brain and external devices, garnering interest due to their potential to transcend human physiological limitations. Decoding and utilizing information about electric potentials generated by the brain is the most prevalent method for implementing BCI (Brumberg et al., 2010). As research into electroencephalograms (EEG) advances, BCI can perform an increasing number of control functions. For instance, Liu et al. (2019) proposed a BCI-based teleoperation strategy for a dual-arm robot with multi-fingered hands handling a common object. Abiri et al. (2020) used imagined body kinesthetics to control a computer cursor. Na et al. (2021) developed an embedded lightweight steady-state visual evoked potential (SSVEP)-BCI electric wheelchair through in-depth SSVEP research, yielding promising experimental results. Willett et al. (2021) achieved previously science-fictional technologies, such as writing with thoughts. A BCI not only enable humans to control external devices subjectively but also facilitate a deeper understanding of ourselves, assisting medical professionals in monitoring patient sleep status (Michielli et al., 2019), evaluating newborn health (Ansari et al., 2019), classifying emotions (He et al., 2020), and obtaining subject status through P300 event-related potential analysis (Banea et al., 2020).

Electroencephalogram acquisition methods are primarily invasive and noninvasive (Ko et al., 2009; Jiang et al., 2018), both of which can use electrodes. The volume of acquired EEG information significantly impacts BCI performance. There are two main approaches to increasing information acquisition: one is to augment the number of EEG acquisition channels. For example, Ma et al. (2018) used 19 channels for EEG analysis (Hefron et al., 2017); Xu and Plataniotis (2016) utilized 32 channels to differentiate affective states; 64 channels were employed for motor imagery (MI) classification, and in some cases, 256 channels were used (Baltatzis et al., 2017). However, increasing the number of EEG acquisition channels yields more information but also prolongs experiment preparation time. This may alter subjects’ mental states, affecting study results and contradicting the convenience and lightweight requirements for broader BCI applications. As the number of EEG acquisition channels grows, the likelihood of channel damage and displacement also rises exponentially. While noninvasive devices can be corrected if detected promptly, invasive acquisition devices are difficult to adjust without causing secondary harm to subjects. Consequently, experiments may be halted due to channel damage or data loss, prompting the use of interpolation or tensor methods to supplement missing information (Sole-Casals et al., 2019). The expectation maximization-based Kalman filter can also estimate missing model signals (Gunnarsdottir et al., 2019). The second approach is to optimize EEG acquisition location and quantity. For example, Zhou et al. (2019) proposed a channel selection algorithm that could automatically choose the minimal electrode subset for a specific subject, achieving satisfactory classification performance using only 5–8 channels of EEG data. Li et al. (2020) introduced a recurrent convolutional neural network model for intention recognition by learning decomposed spatiotemporal representations and used activation mapping visualization technology to select channels with the highest information content, maintaining 92.31% intention decoding performance while reducing nearly half of the acquisition channels. However, optimal channel locations vary across tasks, and actual effects differ among individuals. Therefore, promoting BCI use requires superimposing optimal channels under various circumstances, complicating channel reduction. Simultaneously, channel reduction and layout adjustment necessitate recalculating and retraining the original classification and operation model parameters, hindering the ongoing development of existing BCI systems. Consequently, the challenge in promoting BCIs is reducing the number of required acquisition channels while obtaining more information and avoiding the need to adjust optimal EEG acquisition channels based on the operator and classification task.

Fundamentally, EEG signals in BCIs are collected via electrode channels, with the potential and timeline of each channel forming a one-dimensional (1D) time series. MI can be classified using common spatial pattern (CSP) or independent component analysis (ICA) methods (Ghanbar et al., 2019; Wu et al., 2020), while the wavelet transform (WT) (Geng et al., 2022) enables more detailed EEG analysis and application. In recent years, the rapid development of hardware has led to the emergence of deep learning, which has naturally combined with BCI. Deep learning architectures such as convolutional neural networks (CNN) and recurrent neural networks (RNN) play a crucial role in BCI development (Craik et al., 2020), accelerating its advancement. Using deep learning for MI classification typically achieves accuracies exceeding 80% or even 90% (Lu et al., 2017; She et al., 2019). The choice of time series processing architecture influences BCI system application performance. Researchers have conducted numerous studies to reduce the number of EEGs required for BCI device utilization. For instance, Corley and Huang (2019) implemented super-resolution (SR) based on generative adversarial networks (GANs) to decrease EEG device channel number requirements. Li et al. (2022) proposed an improved Wasserstein generative adaptive network (WGAN) for generating EEG samples in virtual channels, improving sample action classification accuracy. Svantesson et al. (2021) demonstrated that using neural networks to restore or upsample EEG signals was a viable alternative to spherical spline interpolation. Kwon et al. (2019) employed SR technology with deep CNN, proving that exploring various brain dynamics might be possible even with a small number of sensors. Tang et al. (2022) proposed Deep-EEGSR, restructuring corresponding high-resolution (HR) acquisitions through an end-to-end SR process. The Transformer, introduced in 2017, is commonly used to address time series problems such as language recognition (Vaswani et al., 2017). Its attention mechanism emphasizes key aspects of computer learning, achieving nearly overwhelming advantages in speech and image recognition compared to other models (Wang et al., 2020; Chen et al., 2021), becoming a research focal point across various fields. However, the requirement for 10-plus or hundreds of GPUs and datasets with tens of thousands of entries discourages small research teams. Unfortunately, BCI device applications are more personal, making collecting tens of thousands of samples nearly impossible. As a result, the modeling compatibility between BCI and Transformer is poor, with few studies combining the two. Examples include Bagchi’s EEG-ConvTransformer network, achieving high classification accuracy in five different visual stimulus classification tasks (Bagchi and Bathula, 2022), and Jia’s Transformer-based MI classification with good accuracy, applicable to various motion classification scenarios (Jia et al., 2022). The difficulty of utilizing new models over time inevitably hinders BCI development. In 2019, the Informer, based on the Transformer, emerged (Zhou et al., 2021). Guided by the proposed self-attention distilling operation, Informer achieved long-term data prediction for electricity consumption planning. Most importantly, Informer significantly reduced hardware requirements for users when employing Transformer, providing access to small research teams.

In response to the issues above, this study utilized the Informer as a foundation and combined it with BCI device usage to propose an EEG-Completion Informer (EC-informer). Compared to the 1,096,192 samples (iterations of 50 epochs) used in Corley and Huang (2019) and the 5,144 h of data (iterations of 60–100 epochs) used in Svantesson et al. (2021), the EC-informer required only a small number of samples (288 samples, approximately 27 min of data) and 15 epochs to train a satisfactory model. The model’s self-attention distilling operation also reduced hardware requirements for training, enabling training and application on portable devices. As a channel completion model, the EC-informer can establish virtual channels at target locations simply by inputting a limited number of EEG channels. It can also maintain relatively comprehensive EEG information and adapt to BCI application models requiring additional channels. To verify the effectiveness of the EC-informer, several experiments were conducted for analysis: (1) Data from multiple subjects were used to confirm the feasibility of the EC-informer. (2) The impact of the distance between input and target channels on the virtual signal was examined. (3) The performance of the EC-informer was assessed when obtaining EEGs from various acquisition locations. (4) The retention degree of the EC-informer was evaluated in extreme layouts. (5) The applicability of transferring the EC-informer to different subjects was verified. (6) Using a simple classification network, the accuracy of the complementary information obtained by the EC-informer was found to be only slightly lower compared to the real information. (7) The stability of the EC-informer was confirmed using our dataset. The primary innovation of this study is the proposal of the EC-informer, which can reduce the number of actual EEG acquisition channels and complete them with virtual channels. The high information retention of the complementary signal and low hardware requirements could facilitate the promotion of BCI devices.

## 2. Materials and methods

### 2.1. Data and processing

**FIGURE 1:**
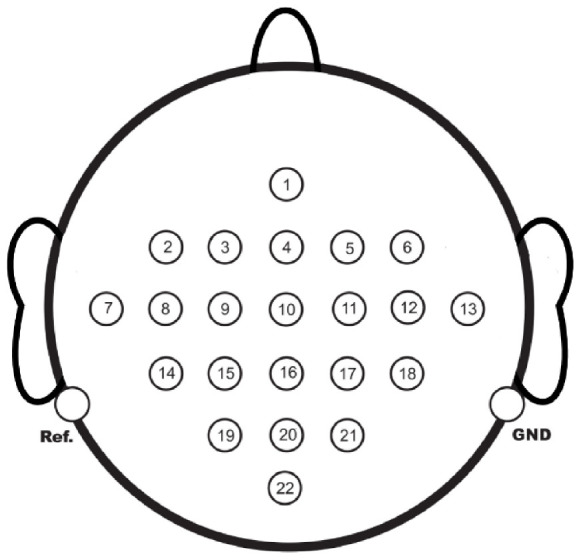
Electrode arrangement of the data set, a total of 22 channels were used.

The dataset utilized in this study was the 2008 2a MI-EEG dataset from the BCI Competition (Tangermann et al., 2012). The dataset employed 22 Ag/AgCl electrodes to collect EEGs from 9 subjects in a non-embedded acquisition mode. The spacing between adjacent electrode channels was 2.5 cm, and the sampling frequency was 250 Hz. The channel distribution is illustrated in Figure 1.

Two sets of experiments were conducted for each subject, serving as training and test sets, respectively. Prior to each experiment set, a 5-min signal recording was conducted to estimate the impact of the electrooculogram (EOG). Following the prompts, subjects were asked to complete four types of imagination tasks, specifically, imagining the movement of the left hand, right hand, both feet, and tongue. Each dataset’s recording time was approximately 2,700 s, resulting in 288 experiments. The primary focus of this study was to use the EC-informer to add virtual channels or complete poor channels, i.e., supplement EEG data. Consequently, sample classification performance was not considered; rather, the obtained virtual or completed EEG was compared to the real information for evaluation.

Brain-computer data is susceptible to external data interference, and EEG information typically falls within the frequency range of 2–40 Hz. Therefore, bandpass filtering is necessary for preprocessing EEGs to eliminate interference from power frequency and excessively high frequencies. Moreover, since the magnitude of the acquired data samples obtained was 10^–6^V, the data needed further processing to adapt them to the EC-informer structure. In this study, the EEG of each channel was standardized and normalized, ensuring the amplitude of the EEG signal ranged between 0 and 1.

### 2.2. EEG-completion-informer

As deep learning technology advances, numerous network models have emerged, such as convolution, RNN, and long-short-term memory (LSTM), which are applicable to tasks like image recognition, behavior recognition, machine translation, and time series forecasting. Recently, the Transformer, composed of multiple layers of encoders and decoders, has gained significant popularity. Each encoder consists of two sublayers: a multi-head self-attention mechanism and a fully connected feedforward network. Compared with the encoder, the decoder adds a masked multi-head attention layer. Thanks to its exceptional mechanism, the Transformer has exhibited remarkable performance across various fields, but it demands more computer hardware resources compared to traditional models. To address this issue, Zhou et al. (2021) introduced the self-attention distilling operation within the Informer structure, significantly reducing the model’s data volume and the Transformer’s hardware requirements. Furthermore, the generative-style decoder in Informer can produce a long sequence output while simultaneously avoiding cumulative error propagation during the inference phase, making it highly effective in time-series forecasting.

Informer employs a global timestamp to analyze the effects of hours, days, weeks, and even festivals on the data, yielding excellent results. This study aimed to create virtual EEG acquisition channels or complete missing ones. The data’s coordinate axis corresponds to the EEG acquisition moment. Although the sample’s specific temporal location throughout the time period or the type of action performed can be used as additional timestamps, they hold little significance for EEG channel data acquisition. This is because EEG signals are nonstationary, highly random, and abrupt. In short and irregular experiments, factors such as age, date, and signal acquisition time can be disregarded. Moreover, while macroscopic human behavior can be somewhat predicted, EEG signals are a combination of external factors (e.g., vision, touch) and subjective factors (e.g., human thinking), constantly changing. Consequently, forecasting EEG signals is extremely challenging, and predicting real-time EEG signals from self-aware humans is nearly impossible. This study employed known EEGs to complete unknown BCI channels rather than predicting future signals, rendering the direct use of Informer unsuitable. Informer was modified to develop EC-informer, which demonstrated considerable advantages in augmenting virtual BCI channels and supplementing poor channels to achieve channel data completion.

In this study, based on the test content, several channels were chosen as known channels, while the remaining channels were deemed unknown, with no electrodes installed in practical applications. Instead, the EC-informer was employed to acquire output EEG information from the (virtual) channel at a specific location for analysis and utilization. Alternatively, if channel data were damaged or information was lost due to external factors, the EC-informer could be utilized to complete the BCI channel. In the subsequent sections of this study, the information obtained from the virtual channel using the EC-informer is referred to as the complementary signal, and the corresponding channel is termed the complementary channel.

The input channel of EC-informer is C_i_ ∈ {C_1_, .., C_n_}, where n is the number of input channels; C_p_ is the complementary channel, C_p_∉{C_1_, .., C_n_}; and ${\text{C}}_{\text{p}}^{{}^{\prime }}$ is the finally obtained complementary signal. In comparison to models like RNN and LSTM, Informer disrupts the temporal sequence, resulting in input data that no longer possesses temporal information (Yan et al., 2022). Hence, it is necessary to incorporate relative and absolute positions for each time instance. The Informer employs the point-wise self-attention mechanism and time stamps to adjust the information’s location, utilizing a sinusoidal positional encoding approach, as illustrated in Equation (1).


(1) $$\left\{ \begin{array}{c} {\text{PE}}_{\left(\mathrm{pos},2\,j\right)}=\text{sin}\,\left(\frac{\text{pos}}{{\left(2\,{\text{L}}_{\text{x}}\right)}^{\frac{2\,j}{{\text{d}}_{\text{model}}}}}\right)\\ {\text{PE}}_{\left(\mathrm{pos},2\,j+1\right)}=\cos\,\left(\frac{\text{pos}}{{\left(2\,{\text{L}}_{\text{x}}\right)}^{\frac{2\,j}{{\text{d}}_{\text{model}}}}}\right) \end{array}\right.$$

Sinusoidal time encoding is conducted, allowing for periodic alterations in the timestamp while confining the range between 0 and 1. The computation of PE in the formula is associated with the parity of pos (index information of data within the time series). The value range of pos can encompass the entire time series or be restricted to a local time index. L_x_ is the length of the time series, and d_model_ is the dimension of the input and output vectors of the model, where j ∈ {1, 2,,[d_model_/2]}. The expressions enclosed in brackets serve to differentiate the positions and periods of data points. This encoding technique effectively circumvents issues related to vanishing or exploding gradients while mitigating the effects of overfitting and noise. The relative location information of the input channel is pos_i_ ∈ {0,1, 2,,L−1}, and the location information of the output signal is posj′{ts,ts+1,…,ts+L-′1}. Here, t_s_ is the initial code of the output label, L′ is the output time-series length, and the following conditions exist:


(2) $$\left\{ \begin{array}{c} {\text{t}}_{\text{s}}\geq 0\\ {\text{t}}_{\text{s}}+L^{\prime }-1\leq L-1 \end{array}\right.$$

In essence, the positional information of the label is entirely encompassed by the input sample’s location information, taking into account both the preceding input channel’s impact on the predicted channel and the predicted channel’s effect on the subsequent input channel. This approach enhances the accuracy of the output channel.

**FIGURE 2:**
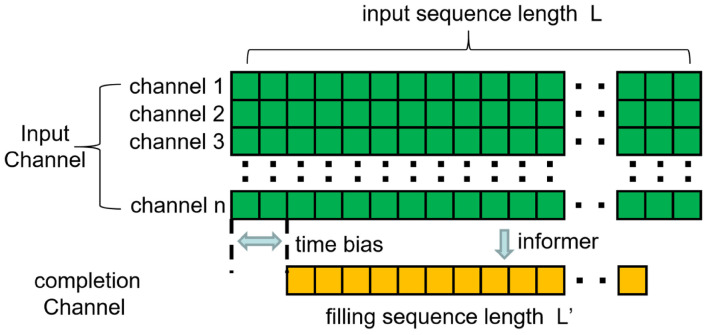
The number of input channels is adjustable, the time series of the target channel label is included in the time series of the input channel, and the starting time is shifted backward.

For the sample at time t, the input data is ${𝒳}^{\text{t}}\,\left\{x_{1}^{\text{t}},\ldots ,x_{{\text{L}}_{\text{x}}}^{\text{t}}|x_{\text{i}}^{\text{t}}\in {\mathbb{R}}^{{\text{d}}_{\text{x}}}\right\}$, where is the channel number of the EEG signal input. The data information outputted by EC-informer is ${𝒴}^{\text{t}}\,\left\{{\text{y}}_{1}^{\text{t}},\ldots ,{\text{y}}_{{\text{L}}_{\text{x}}}^{\text{t}}|{\text{y}}_{\text{i}}^{\text{t}}\in {\mathbb{R}}^{{\text{d}}_{\text{y}}}\right\}$, where d_y_1 is the number of output channels. The association between input channels and complementary channels is illustrated in Figure 2.

**FIGURE 3:**
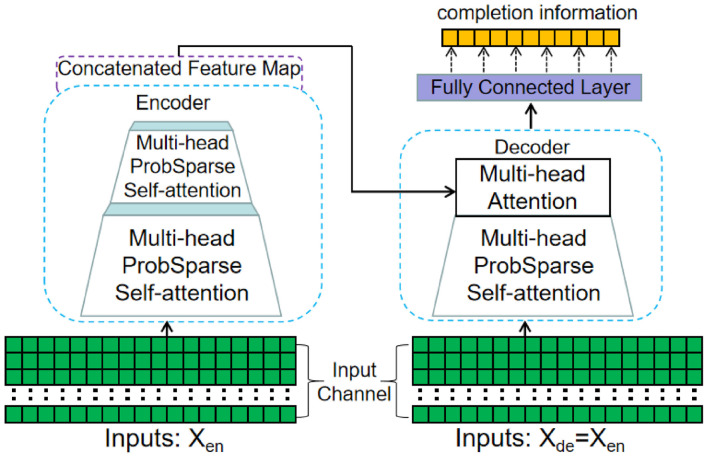
Schematic diagram of the EC-informant structure. It includes two encoder layers and one decoder layer, and uses a self-attention distilling operation to effectively reduce the RAM usage requirements.

Building upon the Informer, the proposed EC-informer consists of a two-layer EC-informer and a single-layer decoder. The detailed structure of the model can be seen in Figure 3.

In the aforementioned figure, the multi-head ProbSparse self-attention block comprises three tensors, namely Query(Q), Key(K), and Value(V), which are computed using three distinct linear layers. Furthermore, Q ∈ R^L_Q_ d^, K ∈ R^L_k_ d^, V ∈ R^V_Q_ d^, where d is the dimension of self-attention. In order to enhance parameter efficiency and decrease the model’s hardware demands, self-attention in this research is derived probabilistically, as illustrated in Equation (3).


(3) $$𝒜\,\left({\text{q}}_{\text{i}},K,V\right)=\sum_{\text{j}}^{{\text{L}}_{\text{V}}}\frac{k\,\left({\text{q}}_{\text{i}},{\text{k}}_{\text{j}}\right)}{\sum_{\text{l}}^{{\text{L}}_{\text{K}}}k\,\left({\text{q}}_{\text{i}},{\text{k}}_{\text{l}}\right)}\,{\text{V}}_{\text{j}}$$

Here, $p\,\left({\text{k}}_{\text{j}}|{\text{q}}_{\text{i}}\right)\,k\,\left({\text{q}}_{\text{i}},{\text{k}}_{\text{j}}\right)/\sum_{\text{l}}^{{\text{L}}_{\text{K}}}k\,\left({\text{q}}_{\text{i}},{\text{k}}_{\text{l}}\right)$, and k(q_i_,k_j_) selects the asymmetric exponential kernel ${\text{q}}_{\text{i}}\,{\text{k}}_{\text{j}}^{\text{T}}/\sqrt{\text{d}}$. As the probability distribution of self-attention is sparse, there is a strong correlation between only certain segments of Q and K. By concentrating on these significant portions of Q and replacing the remainder with the average distribution, the model’s complexity can be effectively reduced. The Kullback-Leibler (KL) divergence is employed to assess the correlation between Q and K in order to identify these important segments of Q.


(4) $$\text{KL}\left(q||p\right)=\text{ln}\sum_{\text{l}=1}^{{\text{L}}_{\text{K}}}\exp\left(\frac{{\text{q}}_{\text{i}}\,{\text{k}}_{\text{l}}^{\text{T}}}{\sqrt{\text{d}}}\right)-\frac{1}{{\text{L}}_{\text{K}}}\sum_{\text{l}=1}^{{\text{L}}_{\text{K}}}\frac{{\text{q}}_{\text{i}}\,{\text{k}}_{\text{l}}^{\text{T}}}{\sqrt{\text{d}}}-{\text{lnL}}_{\text{K}}$$

Upon eliminating the constants, the sparsity measure for the i-th query is defined as:


(5) $$\text{M}\,\left({\text{q}}_{\text{i}},K\right)=\text{ln}\,\sum_{\text{j}=1}^{{\text{L}}_{\text{K}}}\exp\left(\frac{{\text{q}}_{\text{i}}\,{\text{k}}_{\text{l}}^{\text{T}}}{\sqrt{\text{d}}}\right)-\frac{1}{{\text{L}}_{\text{K}}}\,\sum_{\text{l}=1}^{{\text{L}}_{\text{K}}}\frac{{\text{q}}_{\text{i}}\,{\text{k}}_{\text{l}}^{\text{T}}}{\sqrt{\text{d}}}$$

Finally, the softmax function is used to obtain the most important parts of Q:


(6) $$\text{A}\,\left(\bar{\text{Q}},K,V\right)=\text{softmax}\,\left(\frac{\bar{\text{Q}}\,{\text{K}}^{\text{T}}}{\sqrt{\text{d}}}\right)\,\text{V}$$

By identifying the most crucial queries, the model’s complexity can be reduced; however, the length of the time series output from each encoder layer remains unchanged. The addition of encoders leads to an exponential increase in data volume. To some degree, the self-attention distilling operation addresses this issue. The maximum pooling layer employed between two encoders can concentrate essential features, as illustrated in Equation (7).


(7) $${\text{X}}_{\text{j}+1}=\mathrm{MaxPool}\left(\mathrm{ELU}\left(\mathrm{Conv1d}{\left({\text{[X}}_{\text{j}}\right]}_{\text{AB}}\right)\right))$$

where _·AB_ includes the key operations of multi-head probSparse self-attention and attention block; Conv1d (⋅) means 1D convolution on the time series; ELU . is the activation function, and MaxPool. is the maximum pooling layer with a step size of 2. Following the self-attention distilling operation, the cumulative time-series length for each encoder layer is halved compared to the previous layer. This effectively constrains the data volume of model parameters, allowing the model to be trained on GPUs with limited video memory.

The decoder in EC-informer adopts the standard decoder structure in Transformer, and the output result is the channel that needs to be completed. Finally, the mean square error (MSE) is used to calculate the data loss and train EC-informer.

### 2.3. Hardware condition

The primary program was executed on an NVIDIA 3070Ti (8GB memory). Additional configurations comprised an AMD R9 5900HX, 32GB DDR4 RAM, and 256GB M.2 SSD, which were sufficient to meet the research requirements.

### 2.4. Model parameter settings

The dataset employed in this research included 9 subjects, each with a training set (T) and a test set (E). Following model training, the mean square error (MSE) was utilized to assess the complementary signal and the original EEG information. Here, P_l_ is the original signal value at a certain moment, ${\hat{\text{P}}}_{\text{l}}$ is the corresponding value of the complementary channel and L is the length of the data sample. The smaller the value of E, the more closely the complementary signal approximates the original information.


(8) $$\text{E}=\frac{1}{\text{N}}\,\sum_{\text{l}=1}^{\text{L}}{\left({\text{P}}_{\text{l}}-{\hat{\text{P}}}_{\text{l}}\right)}^{2}$$

Moreover, Equation (9) is employed to compute the correlation coefficient between the original signal and the complementary signal, thereby quantifying the congruence of the two signals. xx can be considered as the quantity of information from the original signal retained by the complementary signal. In this context, xx represents the mean value of the original signal and signifies the mean value of the complementary signal. A correlation coefficient of 1 indicates that the two signals are entirely correlated, while a value of 0 implies that the two signals are unconnected.


(9) $$\rho =\frac{\sum_{\text{l}=1}^{\text{L}}\left({\text{P}}_{\text{l}}-\bar{\text{P}}\right)\,\left({\hat{\text{P}}}_{\text{l}}-\bar{\hat{\text{P}}}\right)}{\sqrt{\sum_{\text{l}=1}^{\text{L}}{\left({\text{P}}_{\text{l}}-\bar{\text{P}}\right)}^{2}\times \sum_{\text{l}=1}^{\text{L}}{\left({\hat{\text{P}}}_{\text{l}}-\bar{\hat{\text{P}}}\right)}^{2}}}$$

Including too many EEG channels can create complications and hinder the promotion of BCI, whereas using only 1 or 2 channels can result in insufficient data and reduced accuracy in BCI analysis. To address this issue, the proposed EC-informer selected four EEG channels, and each training session completed one of the lost channels. The input size for both the encoder and decoder was set to 4, while the output size was set to 1. Other parameters used in the study are presented in Table 1.

**TABLE 1:** The remaining parameters.

Description	Parameter	Description	Parameter
Train epochs	3	Dimension of model	128
Batch size of train input data	128	Number of heads	16
Input sequence length of EC-informer encoder	48	Number of encoder layers	2
Label sequence length of EC-informer decoder	24	Number of decoder layers	1
Optimizer learning rate	0.001	Dropout	0.05
Bias	4		

### 2.5. Model training analysis

#### 2.5.1. Applicable objects of EC-informer

**FIGURE 4:**
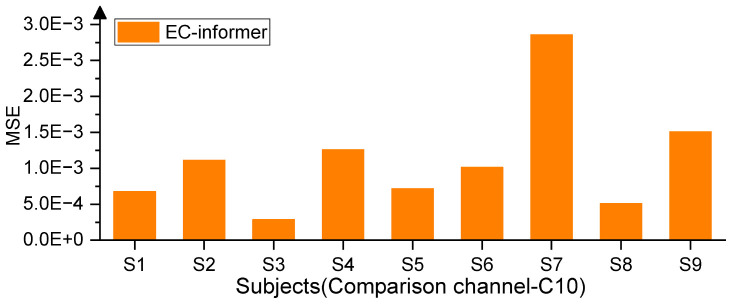
Evaluation results of complementary channels of subjects.

The available EEG acquisition device had 22 channels, with the Cz channel located at the center. Hence, for the first trial, Cz was chosen as the target channel, and the 1st, 8th, 12th, and 20th channels situated 5 cm from the Cz channel were selected as input channels, as seen in Figure 1. The EC-informer was used to complete the missing information of the Cz channel, and the complementary information was obtained. The performance of the proposed approach was evaluated on 9 subjects, and the results are presented in Figure 4.

**FIGURE 5:**
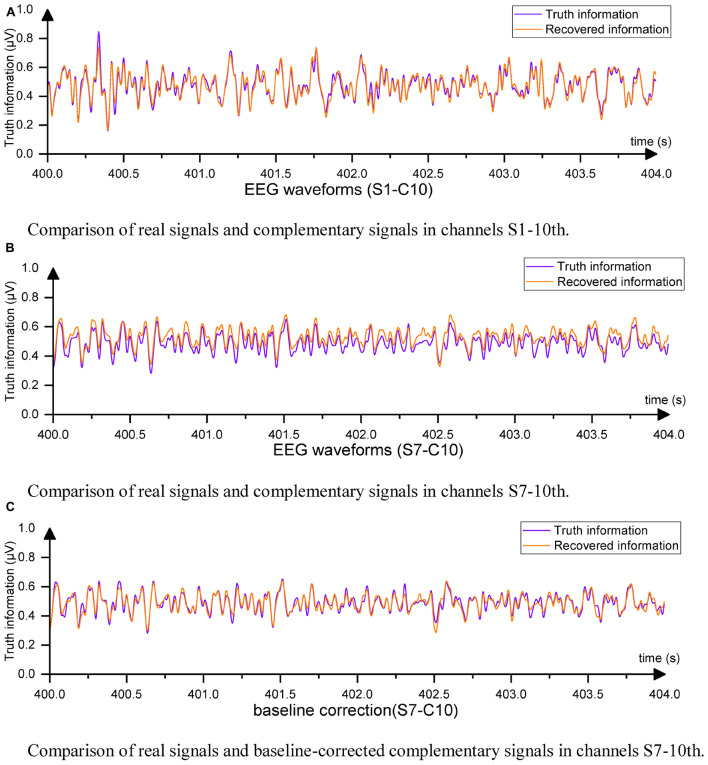
Example of EEG waveformst. **(A)** Comparison of real signals and complementary signals in channels S1-10th. **(B)** Comparison of real signals and complementary signals in channels S7-10th. **(C)** Comparison of real signals and baseline-corrected complementary signals in channels S7-10th.

The EC-informer yielded low MSE values for the complementary signals obtained for all 9 subjects. To provide a comparison of the results, EEG signals from subjects S1 and S7 were selected and plotted in Figures 5A, B.

For S1, the Cz channel data completed using EC-informer with the chosen parameters showed a high degree of data recovery, and the trends of change were almost identical, with only slight differences in amplitude. The correlation coefficient ρ between the two signals was 0.9567, indicating a high level of information retention. For S7, although the MSE was the highest among subjects, the correlation coefficient ρ still reached 0.9334. This result may be attributed to the change in channel placement during the two EEG acquisitions for S7. The supplementary signal showed a slight upward shift, but the waveform was identical to the actual waveform. In most BCI applications, preprocessing, such as mean correction, is performed on each channel, which has little impact on subsequent analysis. Thus, baseline correction was applied to all complementary signals by setting the average values of the output channel data and the Cz channel data to 0. The corrected waveform comparison is shown in Figure 5C.

**FIGURE 6:**
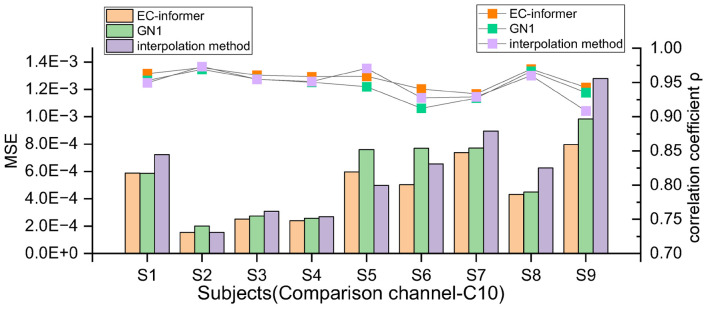
The MSE and ρ of 9 subjects in the 10th channel were compared using EC-informer, GN1, and interpolation method. The results show that the complementary signals obtained using EC-informer have the best performance for most subjects.

All output signals underwent baseline correction, resulting in a high degree of coincidence between the complementary signal and Cz channel data. The new MSE was calculated based on the corrected signals. Furthermore, the MSE between the original information and the interpolated EEG data using Equation (10) was also computed after baseline correction.


(10) $${\text{C}}_{\text{p}}=\sum_{\text{i}=1}^{4}\eta_{\text{i}}\,{\text{C}}_{\text{i}}$$

where i is the channel used, $\eta_{\text{i}}\,1/\left[{\text{d}}_{\text{i}}\,\left(\sum_{\text{i}\,1}^{4}1/d_{\text{i}}\right)\right]$, and d_i_ is the distance from the channel to the target channel. In order to evaluate the effectiveness of EC-informer, it was not only compared with the interpolation method but also with the GN1-model proposed in Ref (Svantesson et al., 2021). However, it was found that the input data length of the original GN1-model (10 s, 2,500 sampling points) did not suit the 2a dataset well, possibly due to too few samples. Hence, the input data length of the GN1-model was changed to 0.4 s (100 sampling points) so that its input channels were the same as those for EC-informer in each comparison. Additionally, the number of training epochs was increased from 60 to 300, and the modified GN1-model showed satisfactory results. The comparison of MSE and the correlation coefficient between the three methods can be seen in Figure 6.

The results indicate that EC-informer had a significant complementary effect on all subjects, as shown by the decreased MSE values. Moreover, the correlation coefficients were greater than 0.93, demonstrating high retention of the original EEG information. Overall, the evaluation results were better than those of the interpolation method and slightly superior to those of the GN1-model, illustrating the high applicability of EC-informer to most operators. Nevertheless, the MSE and correlation coefficients showed variations between subjects, which might be associated with subtle differences in head structure, BCI illiteracy, or factors such as Body Mass Index (BMI), as discussed in Lee et al. (2019) and Gorniak et al. (2022). Nonetheless, these findings imply that there is still value in using EC-informer for channel supplementation.

#### 2.5.2. Relationship between complementary channel and input channel distance

**FIGURE 7:**
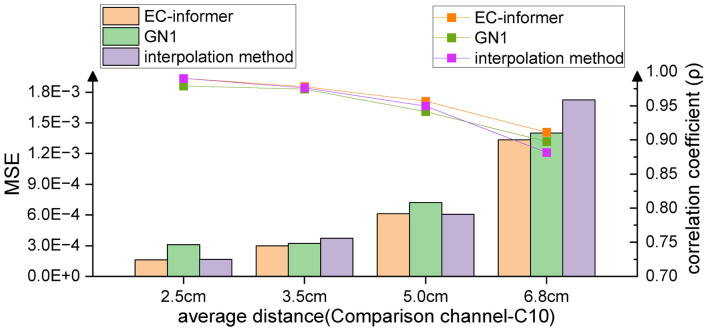
As the distance from the target channel increases, the complementary effects of all three methods decrease, but overall, EC-informer has the best complementary results.

Subject S1 was chosen for a more detailed analysis of the capabilities of EC-informer. The distance between the input channels and the lost channel was found to affect the accuracy of the complementary signal. In order to investigate this effect, the study increased the average distance between the input channels and the lost channel, and evaluated the results, as shown in Figure 7.

**FIGURE 8:**
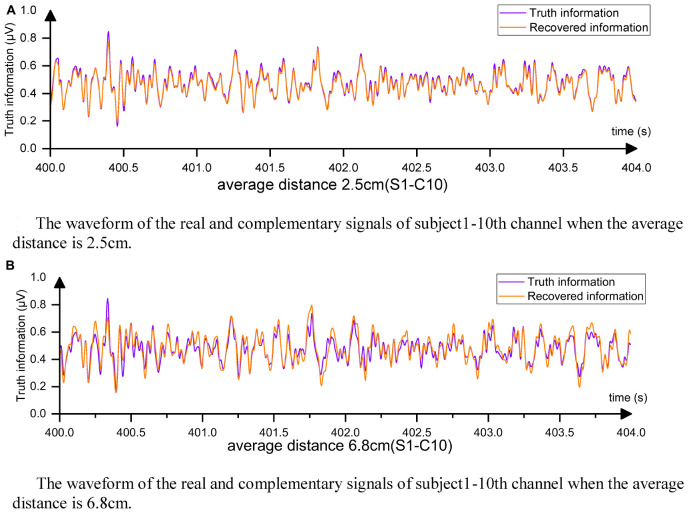
From the figure, it can be observed that the complementary signal and the real signal have a very high degree of overlap. **(A)** The waveform of the real and complementary signals of subject 1–10th channel when the average distance is 2.5 cm. **(B)** The waveform of the real and complementary signals of subject 1–10th channel when the average distance is 6.8 cm.

Figure 8A displays the complementary signal waveform in the test with an average distance of 2.5 cm. As the distance increased, the MSE using the complementary signal decreased and the signal correlation coefficient slightly decreased. In the test with an average distance of 6.8 cm, the combination with the farthest distance to the sample in the dataset, the signal comparison is shown in Figure 8B. The fitting degree using the 1st, 7th, 13th, and 22nd channels to complete the Cz channel was inferior to the 2.5-cm case, but the signal correlation coefficient still exceeded 0.91, indicating excellent fitting performance. Comparing with Figure 7, the advantages of using the complementary signals of EC-informer over the interpolation method and GN1-model were revealed, particularly when the acquisition channels were sparse.

#### 2.5.3. Multi-channel completion of EC-informer

**FIGURE 9:**
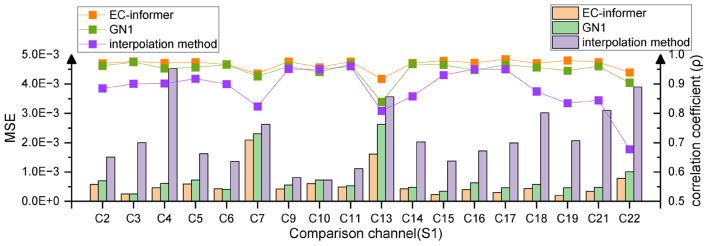
Multi-channel completion effects. Using 1st, 8th, 12th, and 20th channels to supplement the information of the remaining 18 channels of Subject 1, compared to the other two methods, EC-informer retains the most information.

EEG-completion-informer allows for using channels other than the middle channel as complementary channels. In this research, the complementary signals of the remaining 18 channels were obtained using the 1st, 8th, 12th, and 20th channels as input channels. The output MSE for each channel is depicted in Figure 9.

**FIGURE 10:**
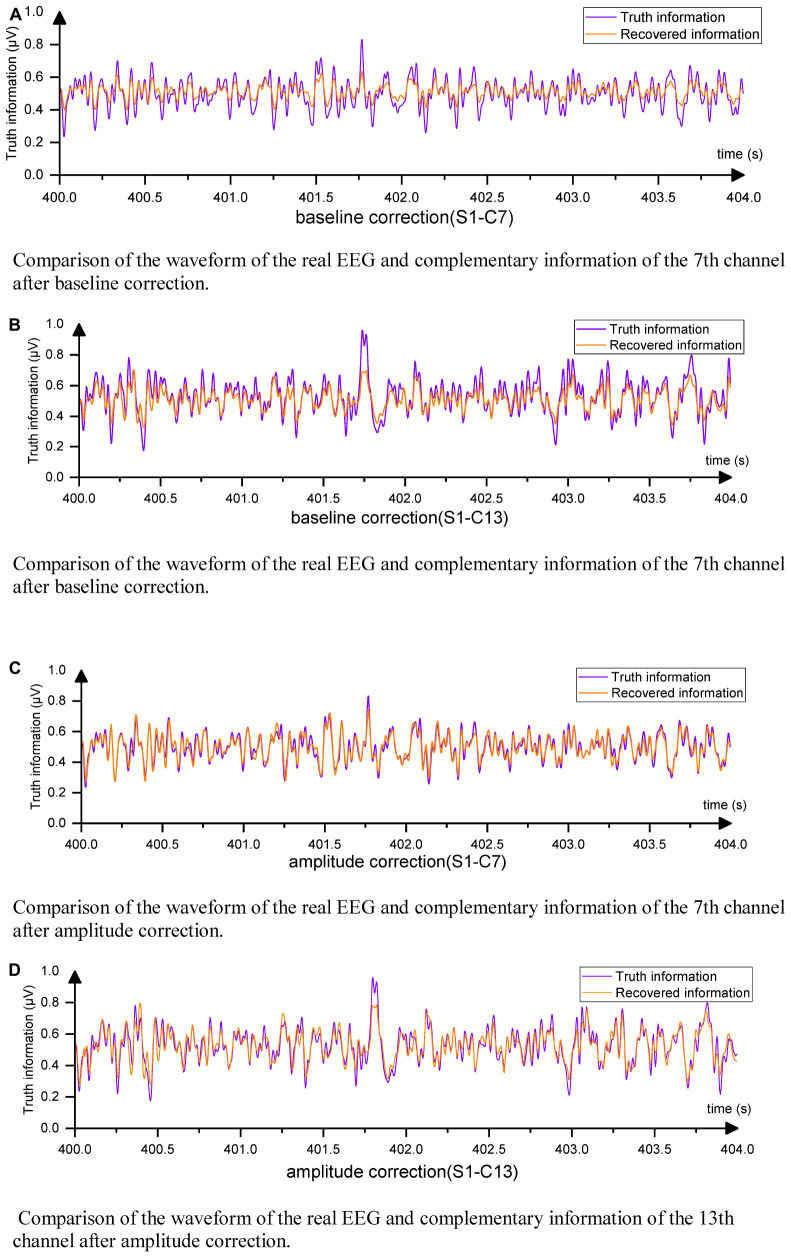
EEG waveforms from direct output. **(A)** Comparison of the waveform of the real EEG and complementary information of the 7th channel after baseline correction. **(B)** Comparison of the waveform of the real EEG and complementary information of the 7th channel after baseline correction. **(C)** Comparison of the waveform of the real EEG and complementary information of the 7th channel after amplitude correction. **(D)** Comparison of the waveform of the real EEG and complementary information of the 13th channel after amplitude correction.

Among the channels, the 7th and 13th channels had the worst MSE values, which may be attributed to their locations at the edges of the EEG acquisition device. The corresponding complementary waveforms are shown in Figures 10A, B.

It can be observed that the differences between the complementary signals were primarily in magnitude, while the trends were similar. By multiplying the complementary channel by a coefficient α, the signal amplitude could be adjusted to approach the original amplitude. Equation (11) details the calculation method for α.


(11) $$\alpha =\frac{\sum_{\text{l}=1}^{\text{L}}\left|{\text{P}}_{\text{l}}\right|}{\sum_{\text{l}=1}^{\text{L}}\left|{\hat{\text{P}}}_{\text{l}}\right|}$$

The obtained new MSE values for channels 7 and 13 were 8.8 × 10^–4^ and 13.17 × 10^–4^, respectively. This reduction in data loss indicated that the amplitude of the complementary signal played a crucial role in affecting the MSE of boundary complementary channels. The processed waveforms are presented in Figures 10C, D, indicating that the complementary signal contained relatively complete EEG change information with a correlation coefficient ρ greater than 0.91. Therefore, only a small amount of information was lost, and the degree of information retention was much higher than that of interpolation and slightly higher than that of the GN1-model in general. This research demonstrated that EC-informer could obtain relatively complete information on multiple channels even with a small number of evenly distributed channels.

As interpolation only completed data between input channels and exhibited poor performance for boundary channels, subsequent research only analyzed EC-informer and did not compare it with the interpolation method.

#### 2.5.4. Signal completion of opposite channels

**FIGURE 11:**
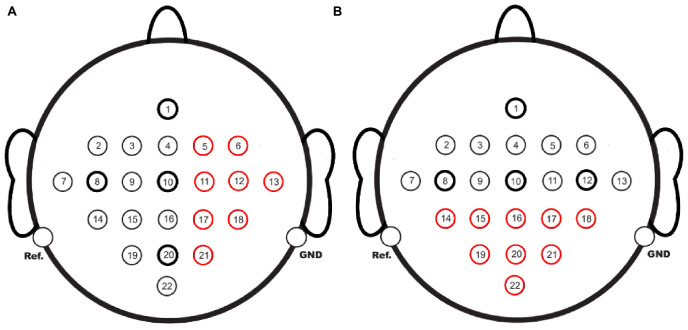
EEG channels to be completed, **(A)** using 1st, 8th, 10th, and 20th channels to supplement the 8 red-marked channels. **(B)** Using 1st, 8th, 10th, and 12th channels to supplement the 9 red-marked channels.

This study also explored the completion of BCI acquisition channels on one side with those on the other side to analyze the effectiveness of EC-informer and GN1-model under extreme cases. The red channels in Figure 11 were taken as the complementary targets. The completion of the channels on the right side using the 1st, 8th, 10th, and 20th channels as input channels is shown in Figure 11A, while the completion of the posterior half channels using the 1st, 8th, 10th, and 12th channels as input channels is shown in Figure 11B. In both cases, the other half channels were used as input.

**FIGURE 12:**
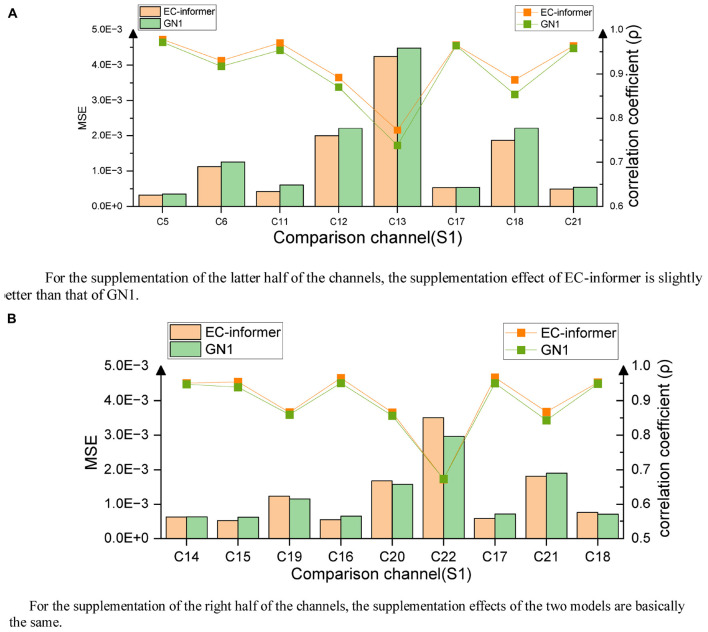
Evaluation results of opposite complementary signals, **(A)** completion results of the right channels, **(B)** completion results of the left channels. **(A)** For the supplementation of the latter half of the channels, the supplementation effect of EC-informer is slightly better than that of GN1. **(B)** For the supplementation of the right half of the channels, the supplementation effects of the two models are basically the same.

After EC-informer training, the evaluation results of the two cases are displayed in Figure 12.

It is evident from the figure that the MSE and the correlation coefficient ρ of the complementary signal were closely related to distance. This phenomenon was primarily due to the different functional divisions of the cerebral cortex in various areas: the further the distance, the greater the functional differences. However, even the farthest channels (i.e., the 13th and 22nd channels) still preserved a significant amount of information for analysis, which demonstrates the outstanding complementary effect of EC-informer.

#### 2.5.5. Migration of EC-informer

Not all subjects can participate in multiple EEG acquisition experiments, which limits the availability of complete data for modeling. Furthermore, due to the significant variations in EEG signals among different individuals, a model that works for one subject may not generalize well to others. Therefore, this study investigated the feasibility of using a trained EC-informer model on other subjects with only one subject’s data available for training. The selected input channels were 1st, 8th, 12th, and 20th, and the target channel for completion was Cz. The model was trained on the data from one subject, and the remaining eight subjects were used for evaluation.

**FIGURE 13:**
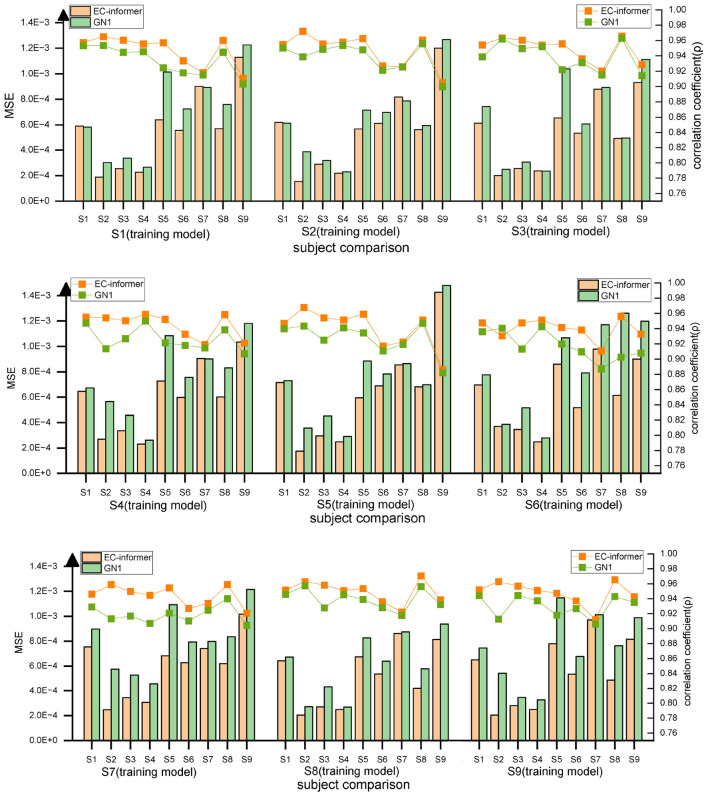
Using transfer to verify the two complementary effects, it can be observed that for most subjects, EC-informer has better complementary effects.

The results, shown in Figure 13, indicate good consistency in MSE and high correlation coefficient *ρ* across different subjects. This suggests that even with only one subject’s data, the model is still effective for completing data for other subjects, demonstrating the excellent generalization potential of EC-informer. Compared to the GN1-model, EC-informer achieved better performance in most cases, highlighting its superiority.

#### 2.5.6. Verification of EC-informer effectiveness

**FIGURE 14:**
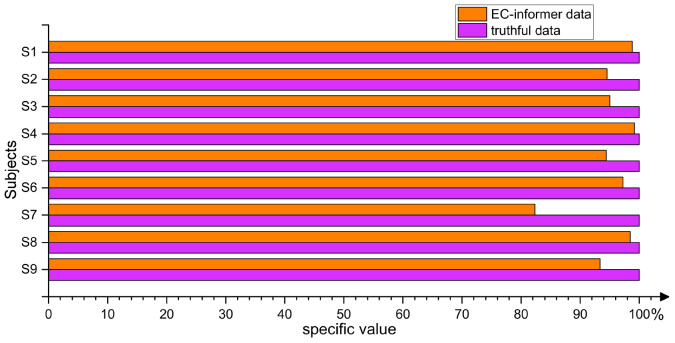
By classifying samples using both the true values and the complementary information obtained from the EC-informer on the validation set, it is proven that using the EC-informer does not lose much information and can still retain most useful signals.

The main objective of using EC-informer was to obtain channels that were not present in the EEG acquisition device or to handle the loss of certain channel signals during abnormal conditions. In order to validate the data obtained by EC-informer, a simple three-layer CNN network was established to classify the four types of actions included in the 2008 2a dataset. The channels used in the classification experiment were the 4th, 8th, 10th, and 20th channels. Real data were used for model training, whereas for the verification set, real data was compared with the complementary signals derived from EC-informer. Taking the accuracy of real data as a baseline, the difference in accuracy between the two verification sets was calculated. The four input channels in the verification set were all complementary signals, emphasizing the gap between the two types of signals. Figure 14 shows the comparison results.

As seen from the figure, the classification accuracy of the complementary signals derived using EC-informer on the verification set decreased slightly compared to that of the real data due to the inevitable loss of local signals in data extrapolation. However, the EEG results obtained using the EC-informer model were still maintained at a relatively satisfactory level, which proved the effectiveness of the EC-informer model in BCI applications. Compared to other methods, EC-informer required fewer samples and iterations to obtain satisfactory data.

#### 2.5.7. Verification of EC-informer stability

To test the stability of EC-informer, inactivity data of 20 min was collected from four healthy subjects. Ten minutes of data were used as the training set, and the remaining 10 min were used as the verification set. Four channels with an average distance of 5.59 cm, namely the 2nd, 6th, 14th, and 18th channels, were used to complement the data of the 10th channel in accordance with the channel layout of 2a. After processing the data as described earlier and training the model with EC-informer, the obtained results are presented in Table 2.

**TABLE 2:** Evaluation statistics.

Subject	S1	S2	S3	S4
MSE	0.00101	0.00174	0.00113	0.00194
Correlation coefficient (ρ)	0.82911	0.86605	0.89126	0.85448

The output results of EC-informer from our dataset were slightly lower than those of the 2a dataset, possibly due to differences in acquisition device accuracy, acquisition environment, and subjects. Nevertheless, the model maintained a low MSE and a high correlation coefficient, indicating good stability and promising application prospects for EC-informer.

#### 2.5.8. Other application examples of EC-informer

EEG-completion-informer has various potential applications beyond the research discussed above. For instance, it can be utilized to identify faulty channels. If the data obtained from a channel of an acquisition device shows significant deviation or lacks correlation with the complementary signal derived from EC-informer, it can be concluded that the channel is damaged or has been affected by additional interference at some point. This can effectively prevent the negative impact of EEG noise on subsequent work. In scenarios where the number of channels available on a new BCI device is limited, EC-informer can be used to generate virtual channels’ EEG, enabling researchers to follow the control strategy of the original BCI without further experiments. This approach reduces researchers’ workload and saves development time, making EC-informer essential for promoting the widespread applications of BCI.

## 3. Results

This study presents the EC-informer deep learning model and its potential applications in BCI devices. Through analyses of the model’s applicability, the relationship between complementary and input channel distances, multi-location channel completion, and migration capability, it was demonstrated that EC-informer could effectively increase the number of acquisition channels for BCI devices. Particularly when only a few acquisition channels are available, EC-informer can provide a satisfactory level of EEG information through complementary effects. It can be used to recover damaged or bad channel data, as well as determine bad channels in experiments and applications. Furthermore, the model’s stability and effectiveness were experimentally verified. These findings suggest that EC-informer has practical significance in BCI research and development.

## 4. Discussion

EEG-completion-informer has a wide application prospect because its ability to complement channels is superior to that of interpolation methods and other network models under many working conditions. Due to the inherent characteristics of the EC-informer, it could be used with a few computing resources, reducing the hardware requirements of the BCI system. A small number of acquisition channels and low hardware demand, together with a high degree of fitting of virtual channel signals, allow the BCI system to maintain high operating accuracy while considering a lightweight and convenient design. In conclusion, the proposed EC-informer has a positive significance in promoting the applications of BCI in daily life.

The EC-informer model is designed for processing long time series data and can be utilized as a data amplification scheme for adding virtual channels in BCI systems to adapt to diverse channel requirements. The model can also be applied to different types of EEG signals that are converted into time series. Furthermore, in systems requiring EEG analysis and showing EEG correlation between channels, EC-informer can be used for channel completion or assessment to enhance system performance.

It is worth noting that the quality of complementary signals provided by EC-informer depends on the input data channels. If there are artifacts or transients in the input channels, they can also appear in the complementary signals and require additional processing techniques such as wavelet transform. When the input channels contain localized fluctuations or spatially limited activities, the complementary signals can partially restore the signals. However, in completely independent scenarios, data recovery is more challenging. There are some limitations to the working conditions of EC-informer. For instance, most existing networks are designed to train for a single channel, and using multiple channels as target channels may result in unstable output and reduced accuracy. Additionally, while a single EC-informer requires less computation and fewer parameters and has low hardware demands, the simultaneous processing of multiple channels can consume more computer resources, limiting its real-time use on portable devices and making it more suitable for offline analysis. Therefore, future research should aim to increase the number of output channels and reduce the hardware requirements for using the model, facilitating its implementation on real-time BCI devices.

## Data availability statement

The raw data supporting the conclusions of this article will be made available by the authors, without undue reservation.

## Ethics statement

Written informed consent was obtained from the individual(s) for the publication of any potentially identifiable images or data included in this article.

## Author contributions

HS and CL designed the EC-informer. HS and HZ analyzed the data and drafted the manuscript. CL and HZ critically revised the manuscript and contributed important. All authors contributed to the article and approved the submitted version.
